# Circadian rhythms regulate osteoclast recycling through gut microbiota-dependent Th17 cell expansion

**DOI:** 10.1016/j.crmicr.2026.100561

**Published:** 2026-01-29

**Authors:** Shuo Ni, Weicong Fu, Lizong Zhang, Zhonghua Zhang, Xiaolin Li

**Affiliations:** a Department of Orthopedic Surgery and Institute of Microsurgery on Extremities,Shanghai Sixth People's Hospital Affiliated to Shanghai Jiao Tong University School of Medicine, Shanghai, China; b Department of Orthopedics, Affiliated Jinhua Hospital, Zhejiang University School of Medicine, Jinhua Municipal Central Hospital, Jinhua, Zhejiang, China; c Animal Experimental Research Center/Institute of Comparative Medicine, Zhejiang Chinese Medical University, Hangzhou, Zhejiang, China

**Keywords:** Circadian rhythm, Time-restricted feeding, Gut microbiota, Th17 cells, Osteomorph

## Abstract

The circadian clock coordinates diverse biological processes to maintain physiological function and homeostasis in mammals under the day-night light cycle. Disruption of circadian rhythms impairs immune and metabolic functions and increases susceptibility to various diseases. Here, we demonstrate that long-term rest-phase time-restricted feeding (TRF), which disrupts circadian rhythmicity, induces bone loss and gut microbiota dysbiosis in male mice. Fecal microbiota transplantation (FMT) from circadian-misaligned feeding donors to germ-free recipients increased Th17 cell populations, thereby promoting the fusion of osteomorphs—a recently identified osteoclast precursor—into mature osteoclasts through the RANKL–RANK–OPG signaling pathway. Collectively, our findings identify a gut microbiota–Th17–osteomorph axis as a critical mediator of circadian disruption–induced bone loss, uncovering a previously unrecognized mechanism by which circadian rhythms regulate skeletal homeostasis.

## Highlights

• Long-term rest-phase time-restricted feeding (TRF) disrupts circadian rhythms, leading to bone loss and gut microbiota dysbiosis in male mice.
• TRF-fed mice exhibit a reduced abundance of *Muribaculaceae* and decreased levels of propionate in fecal samples.
• The abundance of *Muribaculaceae* is positively correlated with bone mass.
• Fecal microbiota transplantation from TRF donor mice to germ-free recipients results in an increased population of Th17 cells.
• The expansion of Th17 cells promotes osteomorph fusion through activation of the RANKL-RANK-OPG signaling pathway.

## Graphical abstract

Circadian rhythms disrupted by misaligned feeding lead to gut microbiota dysbiosis and expansion of intestinal Th17 cells. Microbiota-dependent Th17 cell expansion in misaligned feeding mice drives osteomorphs—a recently discovered osteoclast precursor—to undergo fusion into mature osteoclasts via the RANKL–RANK–OPG signaling pathway, thereby promoting bone loss.

**Figure:**
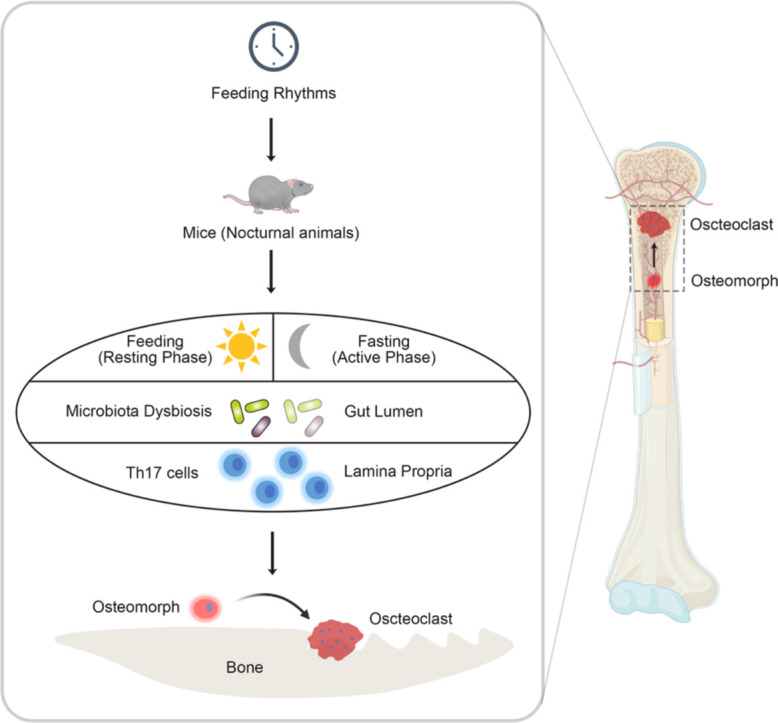

## Introduction

Almost all organisms on Earth exhibit circadian rhythms that synchronize physiological and behavioral processes, such as sleep–wake cycles and feeding patterns (Wier et al., 2010; Stone et al., 2012; Partch et al., 2014; Patke et al., 2020; Brooks et al., 2021). Diurnal animals typically feed during the daytime (or their “active phase”), whereas nocturnal animals eat primarily at night (Brooks and Hooper, 2020). Disruption of circadian rhythms—for instance, through time-restricted feeding (TRF) during the rest phase—can lead to immune cell dysfunction and increased disease susceptibility (Brooks et al., 2021; Yu et al., 2013). Clinical evidence further demonstrates that circadian rhythm disruption adversely affects bone health. Postmenopausal women who work night shifts face a higher risk of bone fractures (Feskanich et al., 2009; Quevedo and Zuniga, 2010), and both men and women with abnormally short or long sleep durations are at increased risk of osteoporosis (Cunningham and Di Pace, 2015; Tian et al., 2015). Collectively, these studies indicate that disruption of physiological circadian rhythms has a detrimental impact on bone health.

The gut microbiota exhibits circadian oscillations that are synchronized with the host’s day–night cycle (Kuang et al., 2019). Conversely, the microbial community also regulates circadian gene expression and influences host immune and metabolic functions (Brooks and Hooper, 2020). For example, the gut microbiota synchronizes with the host circadian clock to regulate innate immunity, thereby preventing *Salmonella typhimurium* infection through activation of immunological circuits in group 3 innate lymphoid cells (ILC3s) (Brooks et al., 2021). Similarly, the microbiota coordinates with the circadian clock to regulate host lipid metabolism (Kuang et al., 2019; Wang et al., 2017).

Bone homeostasis is dynamically maintained through a delicate balance between osteoblast-mediated bone formation and osteoclast-mediated bone resorption (Teitelbaum, 2000). Osteoclasts—the only cells capable of degrading bone matrix—are primarily regulated by RANKL and M-CSF signaling pathways, as well as by immune cells such as Th17 cells (Xiong et al., 2011; Yu et al., 2021). In inflammatory bowel disease (IBD), Th17 cells cooperate with osteoblasts to promote bone resorption (Ciucci et al., 2015), while microbiota-induced Th17 cells migrate to the bone marrow, thereby exacerbating estrogen deficiency–induced bone loss (Yu et al., 2021). Mechanistically, IL-17 secreted by Th17 cells upregulates RANKL expression in osteoblasts and osteocytes, which in turn enhances osteoclast differentiation and activation, ultimately contributing to bone loss (Kotake et al., 1999; Li et al., 2015).

Conventionally, activated osteoclasts were thought to undergo apoptosis after 2–3 weeks, although in some cases they were reported to persist for up to 6 months (Jacome-Galarza et al., 2019; Iqbal and Zaidi, 2021). This raised the question about the behavior and fate of long-lived osteoclasts at resorption sites. Recently, McDonald et al. revealed that mature osteoclasts can undergo fission to smaller, recyclable daughter cells termed “osteomorphs”, which play a key role in osteoclast recycling (McDonald et al., 2021). The authors showed that, under steady-state conditions, osteoclasts undergo fusion in response to M-CSF and RANKL stimulation, but fission occurs following RANKL withdrawal. However, the precise mechanism by which osteomorphs re-fuse to form functional osteoclasts remains unknown.

In this study, we demonstrate that chronic disruption of the circadian clock via long-term rest-phase time-restricted feeding (TRF) induces bone loss through a gut microbiota–dependent pathway involving Th17 cells and osteomorphs. Fecal microbiota transplantation (FMT) from misaligned feeding donor mice into germ-free recipients fully recapitulates the bone loss phenotype. This effect is mediated by enhanced osteomorph fusion into mature osteoclasts, driven by activation of the RANKL/RANK/OPG signaling axis. Collectively, our findings reveal that circadian rhythms, in coordination with the gut microbiota, maintain bone mass by regulating osteomorph fusion dynamics.

## Materials and methods

### Animals

All animal experiments were conducted in strict accordance with the guidelines of the Institutional Animal Care and Use Committee (IACUC) of Zhejiang Chinese Medical University and complied with the ARRIVE guidelines. Ethical approval was granted under Approval Number IACUC-20,211,025-18. Eight-week-old male C57BL/6 mice (specific pathogen-free, SPF grade) were purchased from SLAC Laboratory Animal Co., Ltd. (Shanghai, China). Upon arrival, mice were acclimated for two week before any experimental procedures. Animals were housed in groups of 3–5 per cage in a specific pathogen-free (SPF) facility under controlled environmental conditions (temperature 22 ± 2 °C, relative humidity 50–60 %) with a strict 12:12 light–dark cycle. By definition, zeitgeber time (ZT) 0 corresponded to lights-on at 6:00 a.m. Standard chow diet and water were provided *ad libitum* unless otherwise specified. The total number of animals used in each experiment is reported in the corresponding figure legends. Group sizes were determined based on prior experience and previous publications employing similar experimental designs. For antibiotic treatment, C57BL/6 mice were received 100 mg/L vancomycin (Sigma-Aldrich) in drinking water to deplete commensal gut microbiota. Antibiotic-containing water was freshly prepared and replaced every 2–3 days. Treatment was initiated two weeks prior to the start of the rest-phase time-restricted feeding (TRF) intervention and continued until sacrifice. All animals initially allocated to each group were included in the final analyses unless otherwise stated.

### Time-restricted feeding

Experimental mice were randomly assigned to two groups: *ad libitum* (AL) group and rest-phase TRF (TRF) group. Mice were housed in standard cages provided either *ad libitum* access to chow diet (LabDiet 5P76) or access to a programmable food hopper that opened at ZT0 and closed at ZT8 (8-h feeding window during the light phase). Programmable food hoppers were introduced two weeks prior to the start of the experimental period to allow acclimation to the restricted feeding protocol. Food intake and body weight were monitored weekly throughout the study. After 12 weeks, all mice were euthanized by CO_2_ asphyxiation (fill rate 20–30 % of chamber volume per minute) at ZT6. Fecal samples were collected immediately before euthanasia via stimulated defecation, followed by harvest of tissues and blood for subsequent analyses. The selection of ZT6 for euthanasia was based on previous studies (Li et al., 2020; Zhong et al., 2019), as this mid-light-phase time point minimizes confounding effects of recent feeding or physical activity, thereby enabling more consistent inter-group comparisons of metabolic parameters.

### Germ-free mice and fecal microbiota transplantation (FMT)

Germ-free (GF) C57BL/6 mice were kindly provided by Dr. Lanjuan Li (Zhuge et al., 2025) and were maintained in vinyl positive-pressure isolators at the First Affiliated Hospital of Zhejiang University. Fecal 16S rDNA was routinely assessed by PCR to ensure the GF status. For fecal microbiota transplantation (FMT) experiment, 350–400 mg fresh feces were collected from biological replicates in *ad libitum* and TRF groups donors at ZT6 and immediately resuspended in sterile normal saline. After vigorously homogenizing, the fecal suspension was centrifuged at 400 × *g* for 10 min, and the resulting supernatant—containing the fecal microbiota—was collected for transplantation. For long-term storage, glycerol was added to the supernatant to a final concentration of 10 %, and samples were frozen at –80 °C (Gheorghe et al., 2021; Bokoliya et al., 2021). Recipient mice were gently restrained and orally gavaged with 200 μL of the fecal supernatant using a soft sterile gavage needle attached to a syringe. Gavage was performed once daily for 12 weeks, at the same time each day to minimize circadian variability. In this cohort, 3–4 mice per group were excluded due to dropout following FMT. No adverse events related to the gavage procedure were observed in the remaining animals.

### *In vivo* micro-CT scaning of bone structure

For *in vivo* micro-CT scanning, mice were anesthetized with 1.5–2.0 % isoflurane in oxygen (0.5L/min) to maintain sedation throughout the procedure and facilitate rapid recovery. High-resolution micro-CT imaging was performed using a SkyScan µCT scanner (Bruker MicroCT, Belgium) was used to collect the bone parameter of femur and tibia following the guidelines recommended by Bouxsein *et al*. (Bouxsein et al., 2010). Briefly, images were acquired with an isotropic voxel size of 5 μm from 1000 projections, using an X-ray tube voltage of 70 kVp, current of 200 μA, integration time of 1 s, no frame averaging, and a rotation step of 0.36° over a 180° rotation. Trabecular bone parameters in the distal femur were analyzed in a 1-mm region of interest (ROI) within the trabecular compartment, beginning 100 μm proximal to the distal growth plate, at 5 μm voxel resolution. Cortical bone parameters were evaluated in a 1.5-mm-long mid-shaft region centered at 56 % of the femur length distal to the femoral head, using 10 μm voxel resolution.

### *In vitro* osteoclasts culture

Primary bone marrow-derived macrophages (BMDMs) were isolated from femurs of mice by flushing with MEM culture medium, as previously described (Ni et al., 2020). Cells were cultured in MEM supplemented with 10 % FBS, 20 ng/mL recombinant M-CSF (R&D Systems), and recombinant RANKL (R&D Systems) at concentrations of 0, 50, or 100 ng/mL. Medium was refreshed every 2–3 days, and cells were maintained for 6 days to allow osteoclast differentiation. Mature osteoclasts were then fixed and stained using a tartrate-resistant acid phosphatase (TRAP) staining kit (Sigma-Aldrich). TRAP-positive multinucleated cells (≥3 nuclei) were identified and quantified as osteoclasts.

### Naïve CD4^+^ T cells isolation and Th17 differentiation

Naïve CD4^+^ T cells are isolated from mouse mesenteric lymph nodes (MLNs) using the naive CD4^+^ T Cell Isolation Kit (Miltenyi Biotec) following the manufacturer's instructions. Briefly, MLNs were carefully dissected from surrounding mesenteric connective tissue (typically yielding a chain of 4–8 nodes), placed on the frosted side of a glass microscope slide, and gently dissociated by grinding with a second frosted slide until a single-cell suspension was obtained. The cell suspension was then passed through a 70-μm cell strainer to remove debris, and naive CD4⁺ T cells were subsequently purified following the kit protocol. For Th17 cell differentiation, purified naïve CD4⁺ T cells were cultured in 96-well plates precoated with 10 μg/mL anti-CD3ε and 1.5 μg/mL anti-CD28, supplemented with 20 ng/mL recombinant IL-6 and 5 ng/mL recombinant TGF-β (both from R&D Systems). In addition, to assess immunomodulatory effects, selected wells were additionally treated with 10 μg/mL sodium propionate. Cultures were maintained for 5 days under standard conditions (37 °C, 5 % CO₂). The frequency of Th17 cells (CD4⁺IL-17A⁺) was determined by intracellular cytokine staining and flow cytometry.

### Flow cytometry

Flow cytometry was performed using a Cytoflex LX (Beckman). Live and dead cells were distinguished using 7-AAD. For surface staining, single-cell suspensions were stained with anti-mouse BV510-CD45, PE/Cy7-CD3e and FITC—CD4. For intracellular staining, cells were stimulated with a leukocyte activation cocktail (BD Pharmingen) for 12 h. Anti-mouse BV421-IL-17A antibodies were used after cell fixation and permeabilization using the Intracellular Fixation & Permeabilization Buffer Set (BD Pharmingen). All antibodies used in this study are listed in Table S1.

### Histological analysis

For histological staining, femurs were fixed in 4 % formaldehyde at room temperature and decalcified in a 10 % (w/v) aqueous solution of tetrasodium EDTA. After paraffin embedding, samples were sectioned at a thickness of 4 μm using a microtome (Leica, Germany). Hematoxylin and eosin (H&E) staining, as well as tartrate-resistant acid phosphatase (TRAP) staining, were performed to evaluate histological changes. TRAP staining was used to quantify osteoclast (OC) numbers, whereas H&E-stained sections were scanned using an Aperio ScanScope system.

### Quatitative real-time PCR (qRT-PCR)

For RNA isolation, femurs were carefully dissected to remove all surrounding soft tissues. Bone marrow was then flushed out with sterile PBS and collected, together with the remaining cortical and trabecular bone tissues, for the subsequent RNA extraction. RNA was isolated from both bone marrow and bone tissues following mechanical homogenization. Total RNA was extracted using the RNeasy® Mini Kit (Qiagen) and reverse-transcribed into cDNA using PrimeScript™ RT Master Mix (Takara) following the manufacturer's instrument. Real-time PCR using SYBR Green Master Mix (Vazyme), and relative gene expression levels were calculated using the 2^–ΔΔCT^ method. Primer are listed in Table S2. Notably, the osteoblast-related genes (*Alp, Ocn* and *Runx2*) and osteoclast-related genes (*Trap, Nfatc1, Mmp9, Ctsk* and *Fos*) were measured in bone tissues, whereas the osteomorph-related genes (*Bpgm* and *Fbxo7*), circadian genes (*Bmal1* and *Per1*) and Th17/Treg-related genes (*Il17a, Tgfb1* and *Il10)* were measured in bone marrow.

### Enzyme-linked immunosorbent assays (ELISA)

Mouse serum P1NP and CTX-1 levels were measured using the Mouse Procollagen I N-Terminal Propeptide (PINP) ELISA Kit (Shanghai Jonlnbio Industrial Co., Ltd.) and Mouse CTX-I ELISA Kit (Elabscience), respectively. Bone marrow RANKL levels were measured using Mouse Receptor Activator Of Nuclear Factor Kappa B Ligand (RANKL) ELISA Kit (Shanghai Jonlnbio Industrial Co., Ltd., JL12841). All assays were performed according to the manufacturers’ instructions.

### Bacterial DNA isolation and 16 s gene sequencing

Feces were collected from mice, followed by extraction of bacterial DNA using the MagPure Soil DNA LQ Kit (Magen). The V3-V4 regions of the bacterial 16S rRNA gene were amplified and sequenced by OE biotech Co., Ltd. (Shanghai, China), as previously described (Li et al., 2025). All sequencing data were processed using Vsearch software to generate operational taxonomic units (OTUs).

### SCFA measurement

Feces samples were collected and prepared as previously described and analyzed using an Agilent 1200 series HPLC system to measure the concentrations of acetate and propionate (Smith et al., 2013). Metabolite concentrations were identified and quantified by comparing peak retention times with those of authentic standards.

### Statistics analysis

Data are presented as the mean value with standard error of the mean (SEM), unless otherwise indicated. Two-tailed student's *t*-test and one-way ANOVA followed by post-hoc Tukey's multiple comparison were used to assess statistic differences, unless otherwise specified in the figure legends. The “n” values reported in each figure legend represent biological replicates. A P value < 0.05 was considered statistically significant. All statistical analyses were performed using GraphPad Prism 9.

## Result

### Long-term rest-phase time-restricted feeding induces bone loss by enhancing

#### bone resorption

**Fig. 1:**
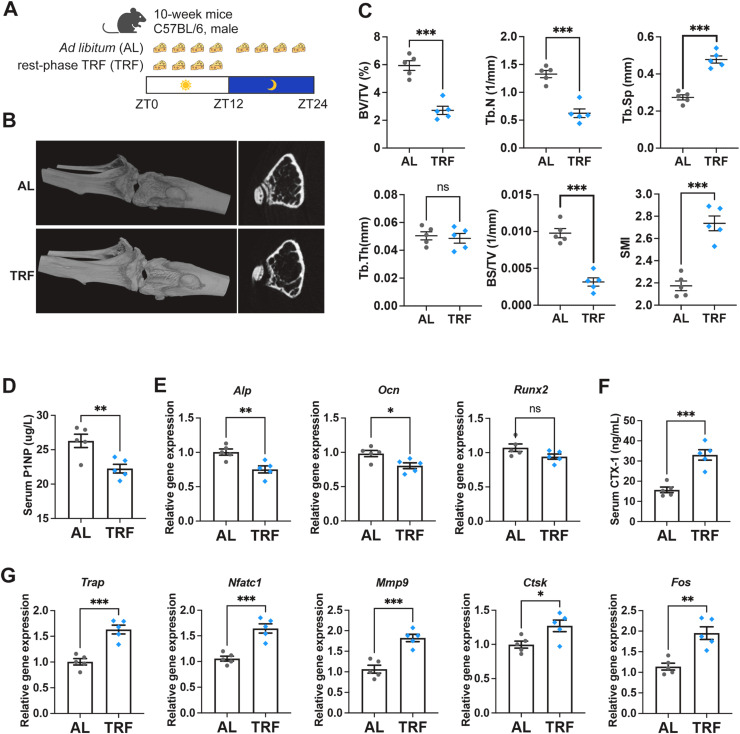
Circadian rhythm dysregulation induced by rest-phase time restricted feeding (TRF) induces bone loss by activating bone resorption. (A) A schematic diagram of mouse experiment. Mice in the *ad libitum* (AL) group were fed with chow diet *ad libitum*, while mice in the rest-phase TRF group were fed with chow diet from zeitgeber time (ZT) 0-ZT8. (B-C) Representative images and statistics quantification of *in vivo* micro-CT scanning. (D-E) Serum P1NP level (D) and relative mRNA expression (E) of osteoblast marker genes *Alp, Ocn* and *Runx2* in the bone tissues. (F-G) Serum CTX-1 level (F) and transcriptional levels (G) of osteoclasts marker genes *Trap, Nfatc1, Mmp9, Ctsk* and *Fos*. Data are presented as mean ± SEM. (*n* = 5 per group; ns, no significance; * *P* < 0.05; ** *P* < 0.01; *** *P* < 0.001).

To investigate the effects of circadian rhythm disruption on bone mass, we established a mouse model of circadian rhythm dysregulation using rest-phase time-restricted feeding (TRF) mouse model (Fig. 1A). After 12 weeks of misaligned feeding, there were no significant differences in body weight (Figure S1A) or weekly food intake (Figure S1B) between the *ad libitum* (AL) and rest-phase TRF (TRF) groups, indicating that the 8-hour feeding window (ZT0-ZT8) provided adequate caloric intake for the mice. Altered expression of the circadian clock genes Bmal1 and Per1 confirmed disruption of circadian rhythmicity (Figure S1C and S1D). In contrast, micro-computed tomography (micro-CT) analysis revealed a marked reduction in trabecular bone mass in the femurs of mice subjected to misaligned feeding (Fig. 1B). Quantitative analysis demonstrated significant decreases in bone volume/tissue volume (BV/TV), trabecular number (Tb.N), bone surface/tissue volume (BS/TV), accompanied by increases in trabecular separation (Tb.Sp) and structure model index (SMI) (Fig. 1C). Notably, these bone phenotypes were observed only in male mice but not in female mice (Figure S1E), indicating a sex-specific effect of rest-phase TRF on skeletal homeostasis.

Generally, trabecular bone mass is primarily determined by the balance between bone formation and bone resorption (Zhong et al., 2023). Accordingly, we next examined biomarkers of osteoblasts (OBs) and osteoclasts (OCs) using ELISA and quantitative real-time PCR (qRT–PCR). The serum concentration of the OB biomarker P1NP P1NP was modestly reduced in misaligned feeding mice (Fig. 1D), and the mRNA expression of OB markers *Alp* and *Ocn* was slightly decreased in the bone tissues (Fig. 1D). In contrast, the serum level of the OC biomarker CTX-1 was markedly elevated in misaligned feeding mice (Fig. 1E), and transcriptional levels of canonical OC biomarkers, including *Trap, Nfatc1, Mmp9, Ctsk* and *Fos*, were substantially upregulated in bone tissues of misaligned feeding mice (Fig. 1G). Together, these results indicate that osteoclast activation induced by misaligned feeding is more pronounced than the accompanying reduction in osteoblast activity. Consistently, these findings support the conclusion that long-term misaligned feeding promotes bone loss primarily by enhancing osteoclast-mediated bone resorption rather than by suppressing osteoblast function.

#### Long-term misaligned feeding reshapes the gut microbiota *in vivo*

**Fig. 2:**
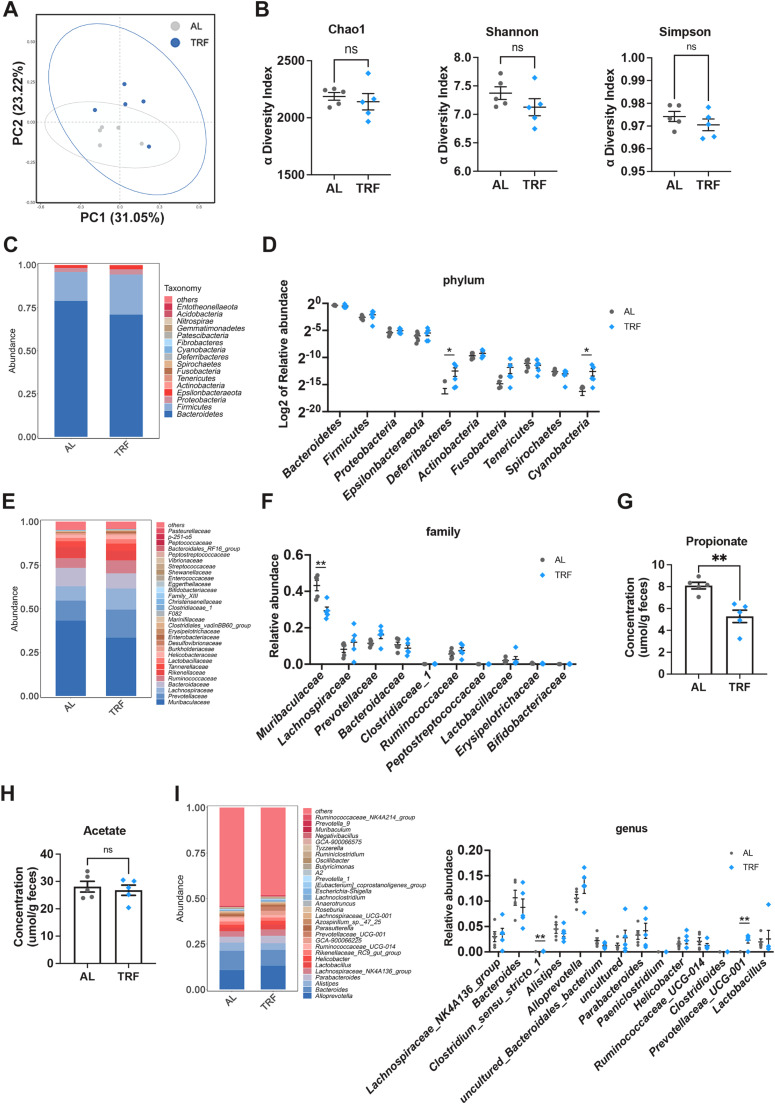
Rest-phase TRF reshaped commensals gut microbiota structure *in vivo*. (A) PCoA analysis of operational taxonomic units (OTU). (B) α-diversity indexs of bacterial 16 s rRNA genes. (C) Group barpolt of the gut microbiota at the phylum level. (D) Top10 abundance of gut microbiota at the phylum level. (E-F) Group barplot and top10 abundance of the gut microbiota at the family level. (G-H) Fecal concentrations of propionate (G) and acetate (H). Data were normalized by the weight of wet feces. (I) Group barplot and top10 abundance of the gut microbiota at the genus level. Data are presented as mean ± SEM. (*n* = 5 per group; ns, no significance; ** *P* < 0.01).

Recent studies have highlighted the critical role of the gut microbiota in regulating bone homeostasis (Guan et al., 2025; Adedigba et al., 2025). To characterize whether long-term misaligned feeding alters gut microbial composition, we collected mouse feces at a specific circadian time point (ZT6) and performed 16 s rRNA sequencing analysis. Although no significant differences were observed in operational taxonomic unit (OTU) numbers or α-diversity between the *ad libitum* (AL) and rest-phase TRF groups (Fig. 2A and 2B), pronounced compositional shifts were detected at the phylum, family, and genus levels. Notably, taxa associated with inflammation, including *Deferribacteres* phylum, *Enterobacteriales* order and *Pseudomonadales* order, were significantly enriched in TRF group (Figure S2A and S2B). At the phylum level, *Bacteroidetes* and *Firmicutes* dominated in both groups, with no significant differences in their relative abundance (Fig. 2C and D). In contrast, the relative abundance of *Deferribacteres* and *Cyanobacteria* was increased in TRF mice, suggesting gut dysbiosis and a pro-inflammatory microbial profile (Fig. 2D). At the family level, the abundance of *Muribaculaceae*, a domininat commensal within the phylum of *Bacteroidetes* known for degrading complex polysaccharides and short-chain fatty acid (SCFA) production, particularly propionate and acetate (Gao et al., 2025), was markedly reduced in rest-phase TRF mice (Fig. 2E and F). This finding suggests that misaligned feeding alters the commensal microbial community and may reduce SCFA availability. Consistently, fecal propionate concentrations were significantly decreased in misaligned feeding mice, whereas acetate levels remained unchanged (Fig. 2G and H). At the genus level, although present at relatively low abundance, *Clostridium sensu stricto 1* and *Prevotellaceae UCG-001* were significantly enriched in the rest-phase TRF group (Fig. 2I), indicating that misaligned feeding induces specific shifts in microbiota rather than a global restructuring of the microbiota. Notably, no significant difference was observed in the relative abundance of *Candidatus Arthromitus* (Figure S2C), a bacterial taxa known to play a critical role in Th17 cell differentiation (Ivanov et al., 2009). Together, these results demonstrate that misaligned feeding reshapes the gut microbial community and reduces SCFA production, particularly propionate.

#### Microbiota-dependent Th17/Treg imbalance contributes to bone loss

**Fig. 3:**
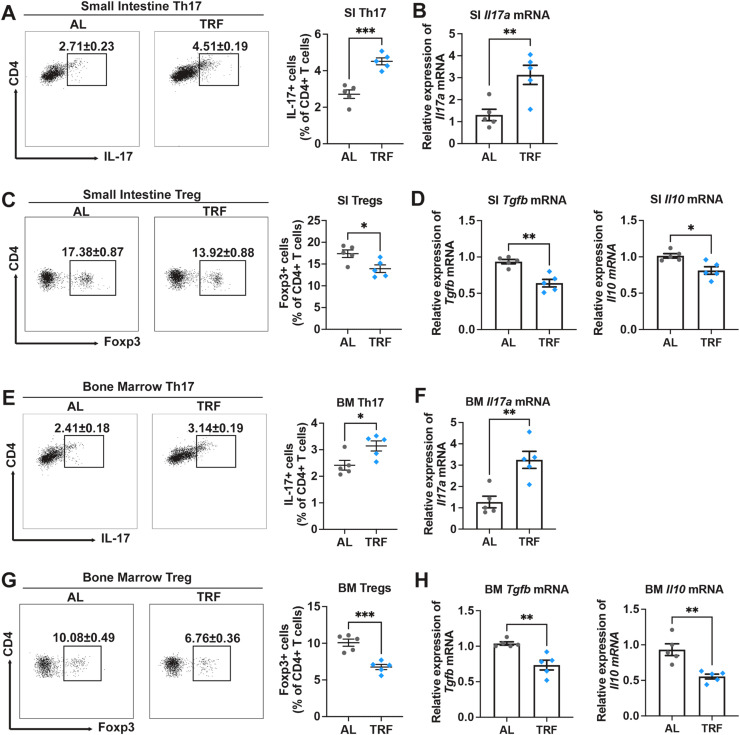
Rest-phase TRF regultates the Th17/Treg balance both in the small intestine and the bone marrow. (A) Dot plots and frequency of Th17 cell in the lamina propria of small intestine. (B) Transcriptional levels of *Il17a* in the small intestine. (C) Dot plots and frequency of Tregs in the lamina propria of small intestine. (D) Transcriptional levels of *Tgfb* and *Il10* in the small intestine. (E) Dot plots and frequency of Th17 cell in the bone marrow tissue. (F) Transcriptional levels of *Il17a* in the bone marrow. (G) Dot plots and frequency of Tregs in the bone marrow. (H) Transcriptional levels of *Tgfb* and *Il10* in the bone marrow. Data are presented as mean ± SEM. (*n* = 5 per group; * *P* < 0.05; ** *P* < 0.01; *** *P* < 0.001).

**Fig. 4:**
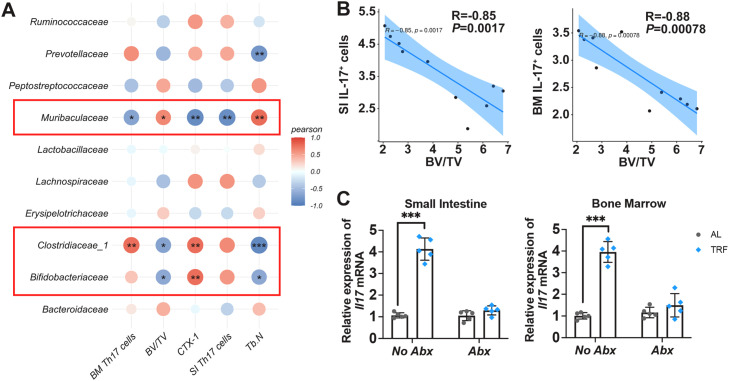
Commensals bacterial are correlated with Th17 cells. (A) Multiple pearson correlation analysis of commensals bacterial, SI and BM Th17 cell frequency and bone mass parameters. (B) Pearson correlation analysis of between BV/TV and small intestine (SI) Th17 cells or bone marrow (BM) Th17 cells. (C) Small intesine and bone marrow transcriptional levels of *Il17a* in antibotics (Abx)-treated mice. Data are presented as mean ± SEM. (*n* = 5 per group; ns, no significance; * *P* < 0.05; ** *P* < 0.01; *** *P* < 0.001).

The immue system plays a pivotal role in the gut-bone axis (You et al., 2025). To determine whether misaligned feeding disrupts immune homeostasis, we analyzed both lymphoid and meyloid immune cell subsets in the intestinal lamina propria by flow cytometry (Shanmugavadivu et al., 2024). Notably, among the immune populations examined, only the proportion of CD4⁺ T cells was significantly increased in rest-phase TRF mice, whereas the proportions of CD8^+^ T cells, neutrophils, macrophages, conventional dendritic cells (cDCs) and cDC1s remained unchanged (FigS3 and FigS4). These results suggest that CD4^+^ T-cell may be a key immune population contributing to misaligned feeding–induced bone loss. Th17 and regulatory T (Treg) cells are two major CD4⁺ T-cell subsets highly enriched in intestinal mucosal surfaces and the lamina propria, and both play critical roles in skeletal homeostasis (Guo et al., 2023; Gaffen and Moutsopoulos, 2020). Compared with AL controls, flow cytometric analysis revealed a significant increase in the frequency of CD4⁺IL-17A⁺ Th17 cells (Fig. 3A), accompanied by elevated *Il17a* transcript levels (Fig. 3B) in the small intestinal of rest-phase TRF mice, indicating that misaligned feeding promotes Th17 expansion in the small intestine. In contrast, the proportion of CD4^+^FOXP3^+^ Tregs was significantly reduced (Fig. 3C), along with decreased expression of *Tgfb* and *Il10* (Fig. 3D), two cytokines closely associated with the anti-inflammatory functions of Tregs. Consistently, similar alterations in Th17 and Treg populations were also observed in the bone marrow (Fig. 3E-H), indicating that misaligned feeding disrupts Th17/Treg balance both locally in the intestine and systemically within the bone marrow.

Furthermore, mutiple pearson correlation analysis indentified three microbial families—Muribaculaceae, Clostridiaceae 1 and Bifidobacteriaceae—were closely associated with bone mass and Th17 cell frequency (Fig. 4A), with Muribaculaceae showing the strongest correlation. Specifically, the relative abundance of Muribaculaceae was positively correlated with bone mass, while being negatively associated with OC biomarker and Th17 cell frequency in both small intestine (SI) and bone marrow (BM) (Fig. 4A). Moreover, Th17 cell percentages in SI and BM were negatively correlated with BV/TV (Fig. 4B), supporting a strong association between Th17 expansion and bone loss. Importantly,antibiotic (Abx) treatment abolished misaligned feeding-induced *Il17a* mRNA expression in both SI and BM (Fig. 4C), indicating that the presence of the gut microbiota is required for misaligned feeding-driven Th17 expansion. Collectively, these data support that a gut microbiota-dependent Th17/Treg imbalance contributes to bone loss in misaligned feeding mice.

#### Fecal microbiota transplantation (FMT) from misaligned feeding mice induces bone loss

**Fig. 5:**
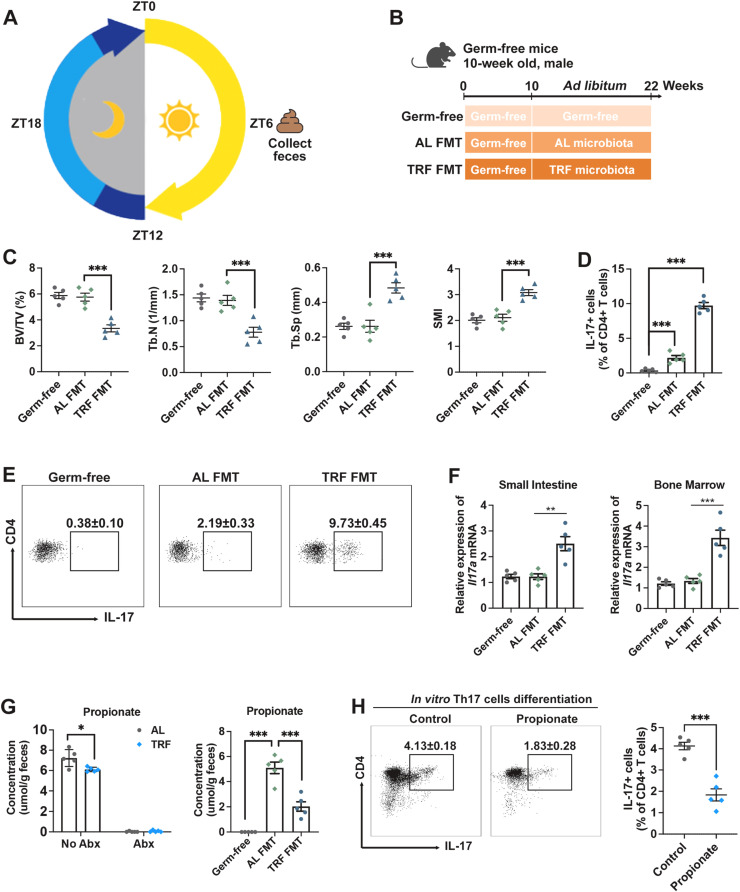
Fecal microbiota transplantation (FMT) from rest-phase TRF mice induces bone loss in germ-free mice fed *ad libitum*. (A-B) Schematic diagram of the FMT experiment in germ-free mice. (C) Quantification of bone mass assessed by micro-CT scanning. (D-E) Representative flow cytometry dot plots and frequencies of Th17 cells in the small intestine of germ-free mice, *ad libitum* FMT mice (AL FMT), and rest-phase TRF FMT mice (TRF FMT). (F) mRNA expression levels of *Il17a* in the small intestine and bone marrow of germ-free mice, AL FMT mice, and rest-phase TRF FMT mice. (G) Fecal propionate concentrations in Abx-treated mice and FMT mice. (H) Representative flow cytometry dot plots and relative frequencies of Th17 cells *in vitro*. Data are presented as mean ± SEM. (*n* = 5 per group; ns, no significance; * *P* < 0.05; ** *P* < 0.01; *** *P* < 0.001).

To further investigate the mechanism by which the gut microbiota mediates misaligned feeding-induced bone loss, fecal microbiota transplantation (FMT) was performed. Feces collected from AL and rest-phase TRF mice at ZT6, the mid-point of the rest-phase, were transplanted into germ-free mice maintained on *ad libitum* feeding (Fig. 5A and B). The mRNA expression levels of the circadian genes *Bmal1* and *Per1* were reduced in the small intestine of recipients receiving fecal transplatation from rest-phase TRF donors (Figure S5), suggesting that circadian rhythmicity in these mice were impaired by FMT. Strikingly, germ-free recipients of rest-phase TRF microbiota exhibited significant bone loss despite continuous *ad libitum* feeding, whereas recipients receiving AL microbiota did not show detectable bone loss (Fig. 5C). Although Th17 cells were nearly absent in the small intestine of germ-free mice, fecal transplantation from both AL and rest-phase TRF donors significantly increased the proportions of Th17 cells in the small intestine (Fig. 5D and E), indicating that FMT induces Th17 cell differentiation in germ-free recipients. Notably, the frequency of Th17 cells in TRF FMT mice was significantly higher than that in the AL-FMT controls (Fig. 5D and E), suggesting that microbiota from rest-phase TRF donors have an enhanced capacity to promote Th17 expansion. Consistently, rest-phase TRF FMT recipients exhibited elevated *Il17a* mRNA levels in both the SI and BM (Fig. 5F), supporting systemic expansion of Th17 cells. In addition, fecal propionate was undetectable in antibiotic-treated and germ-free mice (Fig. 5E and F), confirming that gut microbiota are the primary source of propionate in the intestinal lumen. Although both AL and rest-phase TRF FMT restored fecal propionate production, concentrations remained significantly lower in rest-phase TRF FMT mice compared to AL FMT controls (Fig. 5F). To directly assess whether propionate affects Th17 differentiation, naïve CD4⁺ T cells isolated from mesenteric lymph nodes (MLNs) were cultured *in vitro*under Th17-polarizing conditions (Fig. 5G). Propionate treatment markedly reduced the frequency of IL-17A⁺ CD4⁺ T cells (Fig. 5H), indicating that propionate directly suppresses Th17 differentiation *in vitro*.

#### Misaligned feeding-induced circadian dysregulation promotes osteoclast fusion through RANKL signaling pathway

**Fig. 6:**
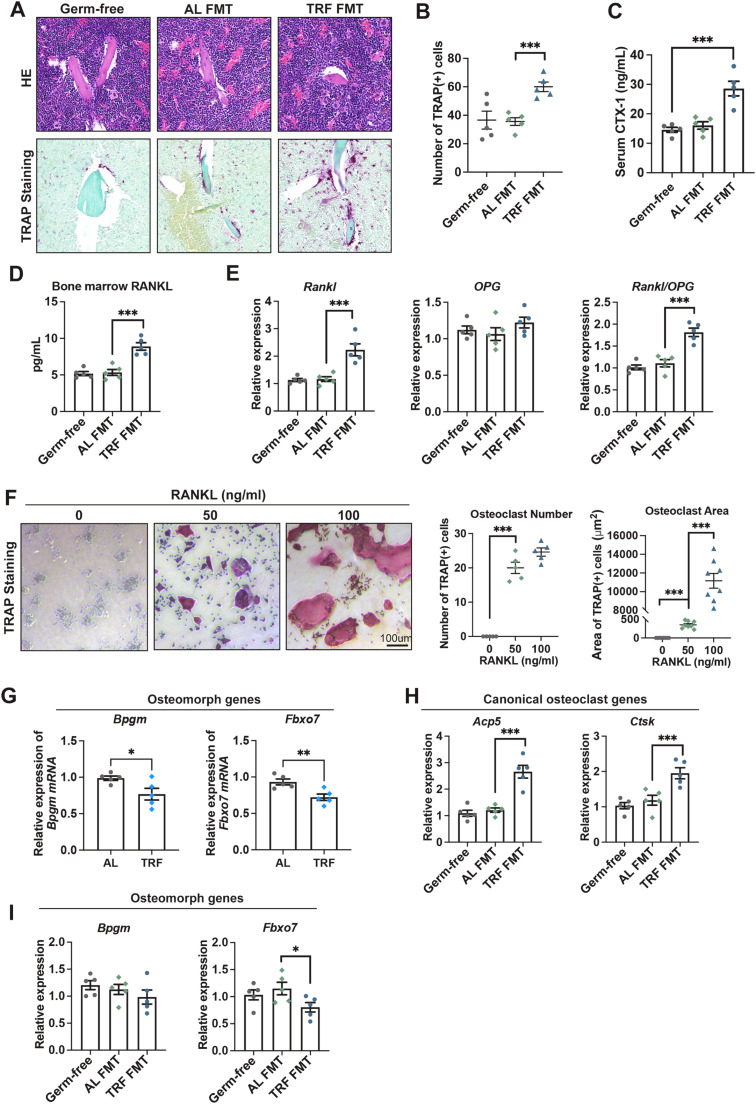
Rest-phase TRF FMT promotes osteoclast fusion through RANKL signaling. (A) H&E and TRAP staining of mouse femur tissues. (B) Quatification of TRAP+ cells number. (C) Serum CTX-1 levels of germ-free and FMT mice. (D) Level of bone marrow RANKL measured by ELISA. (E) Quatification of mRNA expression of *Rankl, Opg* and *Rankl/Opg*. (F) Representative images and quantification of the number and the area of TRAP^+^ osteoclasts *in vitro*. (G) The expression of osteomorph genes Bpgm and Fbxo7 in AL and rest-phase TRF mice. (H-I) Transcriptional levels of canonical osteoclast genes (H) in bone tissues and osteomorph genes (I) in the bone marrow tissues. Data are presented as mean ± SEM. (*n* = 5 per group; * *P* < 0.05; ** *P* < 0.01; *** *P* < 0.001).

To further investigate the mechanisms by which misaligned feeding contributes to bone loss, we performed histological analyses. The number of TRAP^+^ cells was significantly increased in rest-phase TRF FMT mice (Fig. 6A and B), which was further corroborated by elevated serum CTX-1 levels (Fig. 6C). The RANKL–RANK–OPG signaling pathway serves as the principal physiological regulator of osteoclasts (OCs) biology (Kim et al., 2024). Both ELISA and qRT-PCR analyses revealed significantly elevated RANKL levels in the bone marrow of rest-phase TRF FMT mice (Fig. 6D and E). Although OPG expression remained unchanged, the RANKL/OPG ratio was substantially increased (Fig. 6E), indicating activation of RANKL–RANK–OPG signaling under misaligned feeding conditions. Interestingly, *in vitro* experiments demonstrated that higher concentrations of RANKL did not increase osteoclast number, but instead promoted the formation of markedly larger multinucleated OCs compared with those exposed to lower RANKL concentrations (Fig. 6F). Previous studies have shown that OCs can undergo fission into smaller osteomorphs and subsequently re-fuse into OCs at distinct sites (McDonald et al., 2021). To determine whether misaligned feeding affects this dynamic process, we assessed canonical OC-associated genes and osteomorph marker genes by qRT–PCR. The relative mRNA levels of the osteomorph markers *Bpgm* and *Fbxo7* were reduced in TRF mice compared with the AL group (Fig. 6G), suggesting a osteomorph pool under TRF conditions. Consistently, compared with the AL FMT group, the transcript levels of canonical OC genes *Acp5* and *Ctsk* were significantly upregulated in rest-phase TRF FMT mice (Fig. 6H), whereas the mRNA expression of osteomorph marker genes *Fbxo7* was decreased (Fig. 6I), indicating a shift in the balance toward mature osteoclasts at the expense of osteomorphs. Together, these findings suggest that misaligned feeding-induced circadian dysregulation enhances bone resorption by promoting osteoclast fusion while suppressing osteomorph fission.

## Discussion

Here, we report that disruption of circadian rhythm induces bone loss in male mice primarily through gut microbiota dysbiosis. Fecal microbiota transplantation (FMT) from misaligned feeding mice to germ-free (GF) recipients resulted in significant bone loss compared with GF controls, implicating the gut microbiota as a critical regulator of bone homeostasis. Furthermore, our results demonstrate that microbiota-dependent Th17 cell expansion is negatively correlated with bone mass, consistent with previous studies in estrogen deficiency-induced bone loss (Yu et al., 2021; Guo et al., 2023), primary sclerosing cholangitis–associated bone loss (Schmidt et al., 2019) and IBD-induced bone destruction (Ciucci et al., 2015). Importantly, we further identify that misaligned feeding–induced circadian dysregulation–associated bone loss involves osteomorph biology, a recently described osteoclast-derived cell type shown to play a pivotal role in regulating bone remodeling (McDonald et al., 2021). These findings provide new insights into the mechanisms underlying circadian rhythm-disrupted skeletal diseases and suggest alternative therapeutic strategies for bone disorders. Notably, rest-phase TRF-induced bone loss was observed exclusively in male mice, but not in female mice, indicating a pronounced sex-specific effect of circadian disruption on skeletal homeostasis. Consistent with this notion, a recent study by Iiams SE *et al*. demonstrated that appropriately timed TRF prolongs healthspan more effectively in female mice than in male mice, supporting the concept that TRF exerts sex-dependent effects (Iiams et al., 2025). In line with these experimental findings, epidemiological studies have shown that women working night shifts exhibit a significantly increased risk of postmenopausal osteoporosis, with the highest risk observed among women who never used hormone replacement therapy (Feskanich et al., 2009). Together, these observations suggest that estrogen signaling may play an important modulatory role in circadian rhythm–regulated bone homeostasis. However, further investigation is required to elucidate the underlying mechanisms linking sex hormones, circadian regulation, and skeletal remodeling.

Accumulating evidence indicates that disruption of circadian rhythms alter gut microbial composition and increase susceptibility to a variety of diseases (Partch et al., 2014; Brooks et al., 2021; Brooks and Hooper, 2020; Kuang et al., 2019; Wang et al., 2017). However, the impact of circadian dysregulation on skeletal homeostasis—particularly bone mass regulation—has remained largely unexplored. The circadian clock interacts with the gut microbiota through two principal mechanisms: intrinsic daily oscillations and feeding behavior (Brooks and Hooper, 2020). An example of the former is the coordination between the microbiota and the host circadian clock in regulating lipid metabolism (Kuang et al., 2019; Wang et al., 2017). Conversely, altered feeding patterns can reshape microbial rhythmicity, leading to microbiota-dependent metabolic disturbances (Thaiss et al., 2014; Thaiss et al., 2016; Zarrinpar et al., 2014; Leone et al., 2015). In this study, we established a rest-phase TRF model to disrupt circadian rhythms in mice, which are nocturnal animals that normally consume approximately 80 % of their caloric intake during the dark phase under *ad libitum* conditions (Damiola et al., 2000; Arble et al., 2009). Shifting food intake from the active phase to the rest phase induces circadian misalignment and metabolic dysfunctionc (Mukherji et al., 2015; Ye et al., 2024), thereby recapitulating key features of circadian disruption observed in humans. Using this model, we identified gut microbiota dysbiosis characterized by a marked reduction in Muribaculaceae abundance in rest-phase TRF mice. Although Muribaculaceae represent a dominant bacterial family in the murine gut (Lagkouvardos et al., 2019), their role in circadian regulation and skeletal diseases has received limited attention. Recent studies have linked Muribaculaceae to disease severity in colitis (Dong et al., 2023), endometritis (He et al., 2025), and chronic inorganic arsenic exposure (Li et al., 2025). Importantly, Muribaculaceae are capable of producing short-chain fatty acids (SCFAs), particularly propionate, as a fermentation end product, which have been shown to exert protective effects against pathological bone loss (Lucas et al., 2018; Smith et al., 2021). Consistent with our observations several studies have demonstrated that mistimed feeding alters SCFA-producing taxa, including *Firmicutes* and members of the *Ruminococcaceae* family (Bishehsari et al., 2021; Cui et al., 2022). Together, these studies strengthen the reproducibility and contextual relevance of our microbial observations. Accordingly, the reduction in propionate levels observed in misaligned feeding mice may partially explain the associated bone loss phenotype. Nevertheless, Th17 cell differentiation is also intrinsically regulated by the circadian clock (Yu et al., 2013). Therefore, *in vivo* propionate supplementation in misaligned feeding mice would provide a more rigorous approach to directly test the causal role of propionate in regulating Th17 expansion. Due to the scope of the current study, such interventions were not performed, and future studies will be required to address this question. Notably, vancomycin we used in our study has been shown to have an effect on bone mass in early and mature life stages (Lyu et al., 2023; Guss et al., 2017; Cox et al., 2014; Cho et al., 2012). However, bone structural parameters were not assessed in antibiotic-treated mice in the present study. Future experiments will be necessary to determine whether TRF directly affects bone mass under antibiotic-treated conditions. In addition, SCFAs are predominantly produced in the colon, with Th17 differentiation occurs mainly in the ileum. Our findings indicate that colonic-derived propionate is associated with reduced Th17 cell frequencies in the ileum, which may be explained by the entry of SCFAs into the portal circulation, subsequent hepatic processing, and distribution via the systemic circulation to distal tissues, including the ileum. Alternatively, SCFAs may reach the ileum through retrograde diffusion (Boets et al., 2017; Ruppin et al., 1980). Moreover, consistent with previous reports, circadian gene expression in the gastrointestinal tract is markedly reduced in germ-free or antibiotic-treated mice (Wang et al., 2017; Mukherji et al., 2013), supporting the concept of reciprocal regulation between the host circadian clock and the gut microbiota. In line with these observations (Kuang et al., 2019; Wang et al., 2017), demonstrate that circadian disruption–induced microbiota dysbiosis exerts a detrimental effect on bone mass. Finally, longitudinal assessment of bone parameters and circadian gene expression, as well as shorter or longer rest-phase TRF, may provide further insights into the temporal dynamics by which misaligned feeding induces bone loss. Additionally, longitudinal 16 s rRNA sequencing of germ-free recipients following FMT, combined with intermediate measurements of bone parameters, would help delineate the kinetic relationship between microbiota remodeling and skeletal outcomes. These limitations are acknowledged in the present study and will be addressed in future work.

Since the concept of osteoimmunology was first proposed by Arron and Choi in 2000, interactions among the gut microbiota, the immune system, and the skeleton have been increasingly recognized as critical regulators of bone homostasis (You et al., 2025; Arron and Choi, 2000). Our observation of altered CD4⁺ T-cell prompted us to focus on Th17 cells, a specialized lineage of CD4⁺ T cells that is highly enriched at intestinal mucosal surfaces and plays a pivotal role in skeletal homeostasis. Indeed, Th17 cells have been implicated as potent osteoclastogenic activators, whereas regulatory T (Treg) cells exert protective effects on bone mass by restraining excessive immune activation (Guo et al., 2023). Consistent with this concept, Sapra *et al*. reported an expansion of Th17 in ovariectomized mouse models (Sapra et al., 2025). In agreement with these findings, our study demonstrated that the proportions of Th17 cells both in the small intestine and the bone marrow were negatively correlated with bone mass, supporting a pathogenic role of Th17 cells in misaligned feeding-induced bone loss. However, it should be noted that direct evidence for local Th17 expansion within the bone marrow is currently lacking. The increased Th17 population observed in the bone marrow may arise either from *in situ*differentiation or expansion within the bone marrow microenvironment, or from the migration of Th17 cells from the small intestine to the bone marrow. Meanwhile, Th17 and Treg cells constitute only a small fraction of the total CD4⁺ T-cell compartment. Due to technical limitations, we did not characterize other immune cell populations or systematically assess circulating short-chain fatty acid (SCFA) levels in this study. Future investigations incorporating broader immune profiling and metabolomics analyses will be required to fully delineate the immunometabolic signals underlying misaligned feeding–induced bone loss.

Osteomorphs are a recently desciribed cell type first identified by McDonald *et al*. in 2021 (McDonald et al., 2021). These cells undergo fusion to form activated osteoclasts in response to RANKL stimulation and undergo fission upon RANKL withdrawal. Osteomorphs are defined by a distinct transcriptional program comprising 151 gene signatures, which distinguish them from mature osteoclasts, including bisphosphoglycerate mutase (*Bpgm*) and F-box protein 7 (*Fbxo7*). In our study, we observed a reduction in *Fbxo7* expression in rest-phase TRF FMT mice, suggesting enhanced fusion of osteomorphs into activated osteoclasts, thereby contributing to increased bone resorption under conditions of circadian disruption. However, as reported by McDonald *et al*. (McDonald et al., 2021), osteomorphs are difficult to identify and quantify *in vivo* using conventional histological approaches. Accordingly, in the present study, osteomorph dynamics were assessed primarily at the transcriptional level. Future studies employing lineage-tracing strategies and functional assays will be required to more definitively characterize osteomorph behavior and function under misaligned feeding conditions.

In conclusion, our study demonstrates that misaligned feeding modulates skeletal homeostasis through alterations in the gut microbiota. Previous studies have shown that misaligned feeding induces circadian reprogramming, leading to adverse health outcomes (Acosta-Rodríguez et al., 2024; Honzlová et al., 2022). Given that night-shift workers exhibit increased prevalence of metabolic disorders, including insulin resistance and obesity (Brooks and Hooper, 2020; Reid et al., 2014), our findings further underscore the importance of temporally aligned feeding and circadian–microbiota synchronization in maintaining bone health. This effect may be attributed to altered feeding behaviors and impaired coordination between the host circadian clock and the gut microbiota (Kuang et al., 2019; Wang et al., 2017). The observed association among feeding schedules, gut microbiota, and bone homeostasis raises the possibility that circadian-aligned dietary interventions may represent a non-pharmacological therapeutic strategy for preserving or restoring bone mass. In parallel, microbiota-based approaches, including probiotic supplementation, dietary modulation, or FMT, may serve as alternative strategies to suppress pathological osteoclast activation. However, our data support the view that propionate plays a context-dependent regulatory role in microbiota–immune–bone crosstalk, rather than acting as a single determinant of Th17-cell expansion or bone homeostasis. Future studies should investigate whether distinct dietary compositions (*e.g.*, high-fat diet) or specific microbial metabolites modulate bone remodeling under conditions of circadian disruption, thereby providing a more comprehensive understanding of diet–microbiota–circadian interactions in skeletal health.

## Conclusion

Long-term rest-phase time-restricted feeding disrupts circadian rhythmicity, leading to bone loss by enhancing osteoclast activation through increased fusion of osteomorphs into mature osteoclasts via the RANKL–RANK–OPG signaling pathway. The gut microbiota is indispensable for this process, as fecal microbiota transplantation (FMT) from misaligned feeding mice into germ-free recipients recapitulated osteoclast activation and bone resorption through microbiota-dependent Th17 cell expansion. Collectively, these findings proposed a novel framework in which circadian rhythms regulate skeletal homeostasis through a gut-immune-bone axis.

## Ethics statement

All animal experiments were conducted follwing the guidelines of the Institutional Animal Care and Use Committee of Zhejiang Chinese Medical University (Approval Number: IACUC-20211025-18).

## Funding

This work is supported by the 10.13039/501100001809National Natural Science Foundation of China (No . 82​​200​​994 ), 10.13039/501100002858China Postdoctoral Science Foundation (Grant No 2022M712113), Shanghai Post-doctoral Excellence Program ( Grant No 2022428) to S.N.; 10.13039/501100001809National Natural Science Foundation of China (No. 82​​072​​422) to X.L.; and the Research on the Application of Digitalized and Minimally Invasive Hip Arthroplasty Technology supported by Science and Technology Bureau of Jinhua, Zhejiang (Grant No 2024–3-053) to W.F.

## Data availability statement

Sequencing datasets are available at the Gene Expression Omnibus repository. Any additional information in this paper is available on request.

Supplementary Figure 1. Mouse body weight and food intake.

Supplementary Figure 2. LEfSe cladograms identified abundant taxa at different levels.

Supplementary Table 1. Antibodies for flow cytometry.

Supplementary Table 2. Primer sequences for qRT-PCR.

## Declaration of competing interest

The authors declare no conflict of interest.

## Appendix Supplementary materials

Image, application 1
