# Supplementary material for: Circadian rhythms regulate osteoclast recycling through gut microbiota-dependent Th17 cell expansion
Source: Curr Res Microb Sci. 2026 Jan 29;10:100561. doi: 10.1016/j.crmicr.2026.100561 (PMC12906124; doi:10.1016/j.crmicr.2026.100561)
Supplement: Supplementary file 1 [file mmc1.docx]

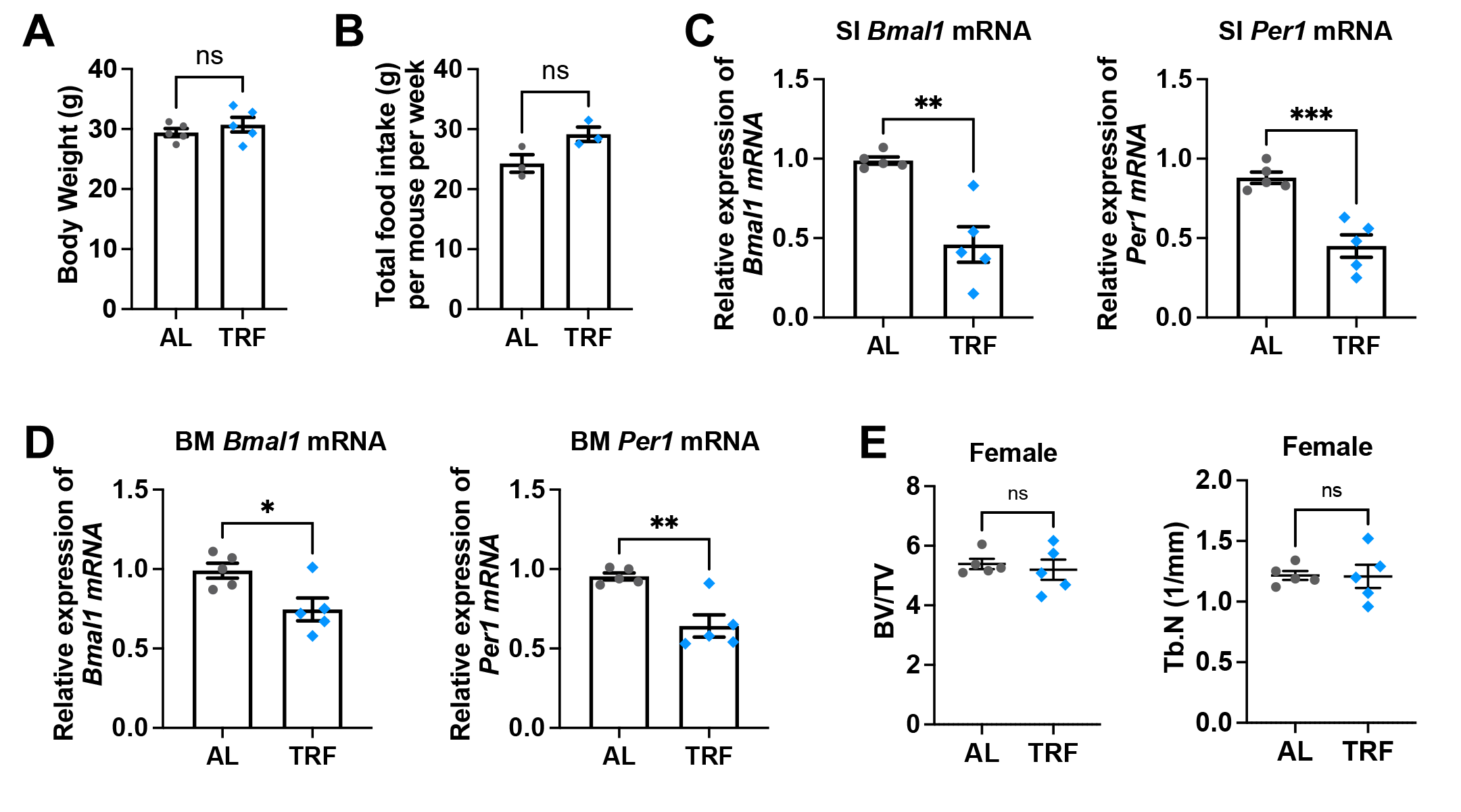


**Figure S1.** Body weight, food intake and bone parameters of mice exposed to rest-phase TRF. (A) Mice body weights. (B) Total food intake. Food consumption was presented as gram per mouse per week. (C) The mRNA expression of circadian genes *Bmal1* and *Per1* in the small intestine. (D) The mRNA expression of circadian genes *Bmal1* and *Per1* in the bone marrow. (E) Bone volume/ tissue volume (BV/TV) and trabecular number (Tb.N) parameters of female mice exposed to rest-phase TRF for 12-week. Data are presented as mean ± SEM. (n=5 per group; ns, no significance; * *P*<0.05; ** *P*< 0.01; *** *P*< 0.001).

**
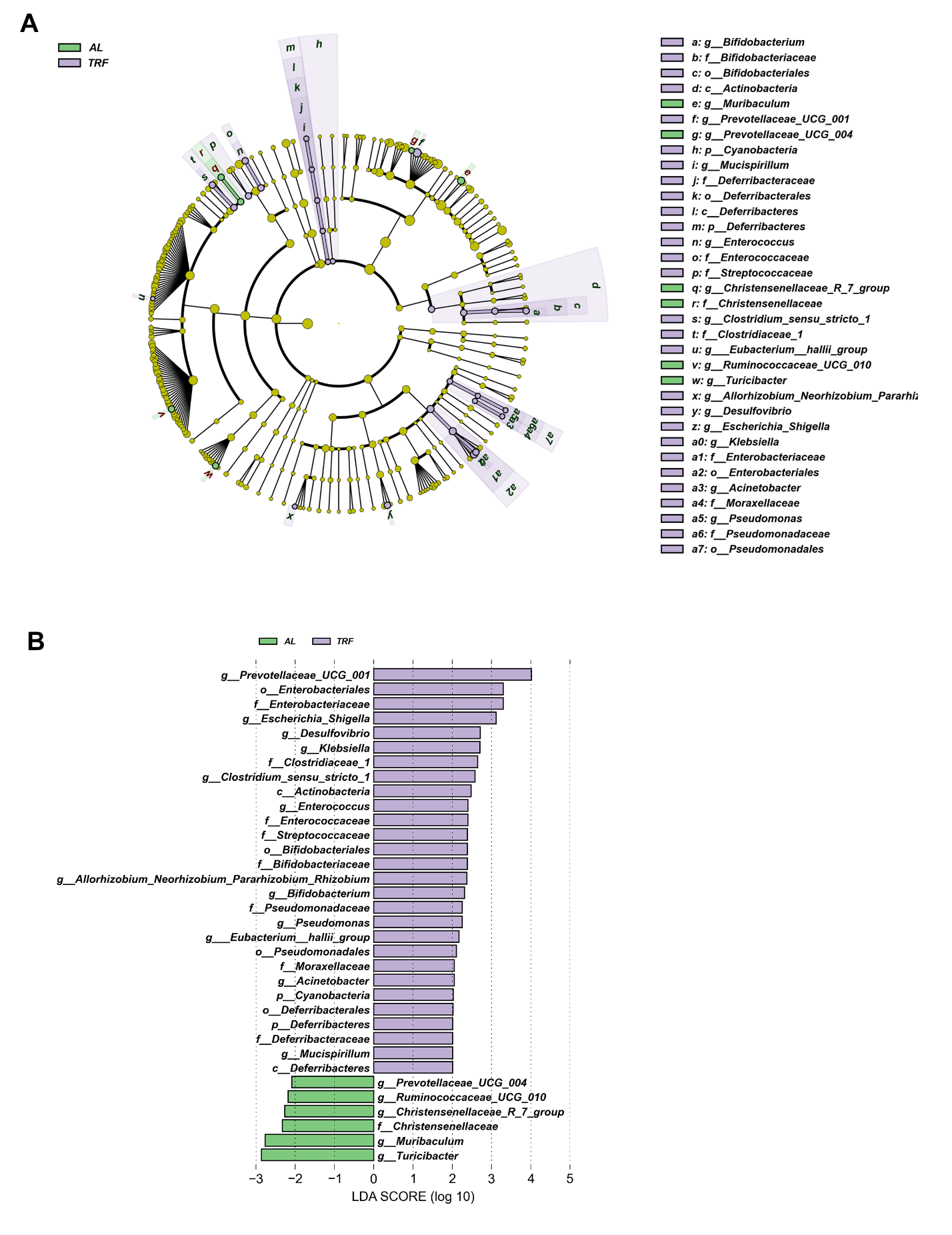
**

**Figure S2.** LEfSe analysis of abundant taxa at different levels. (A) LEfSe cladogram of abundant taxa from AL and rest-phase TRF groups. (B) LDA score histogram analyzing bacterial differences between AL and rest-phase TRF groups.

**
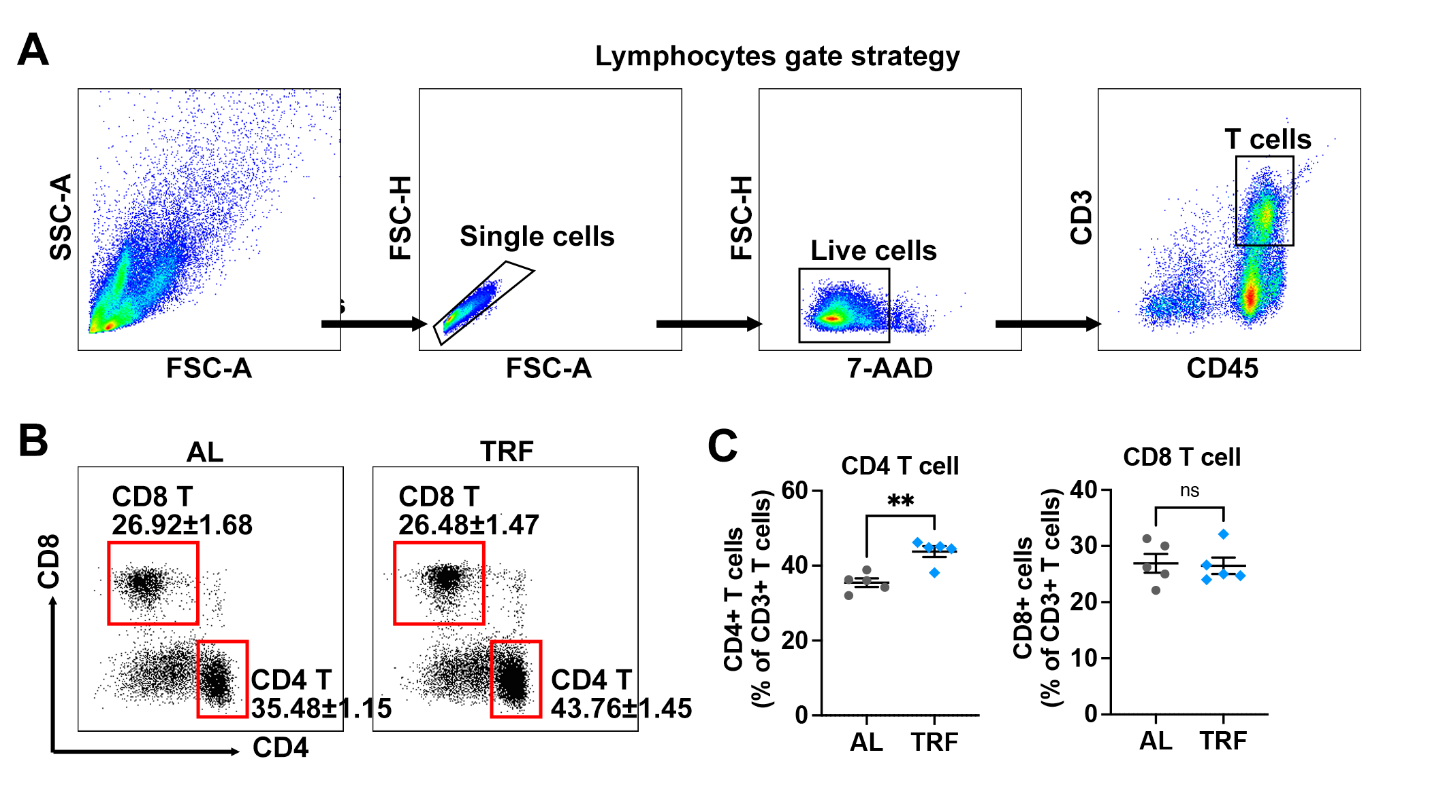
**

**Figure S3.** Lymphoid cells in the lamina propria of small intestine. (A) The flow cytometric gate strategy of Lymphocytes. (B) Dot plots of CD4+ and CD8+ T-cell subsets analyzed by flow cytometry. (C) Frequencies of CD4+ and CD8+ T cells among CD3+ cells. Data are presented as mean ± SEM. (n=5 per group; ns, no significance; ** *P*< 0.01).


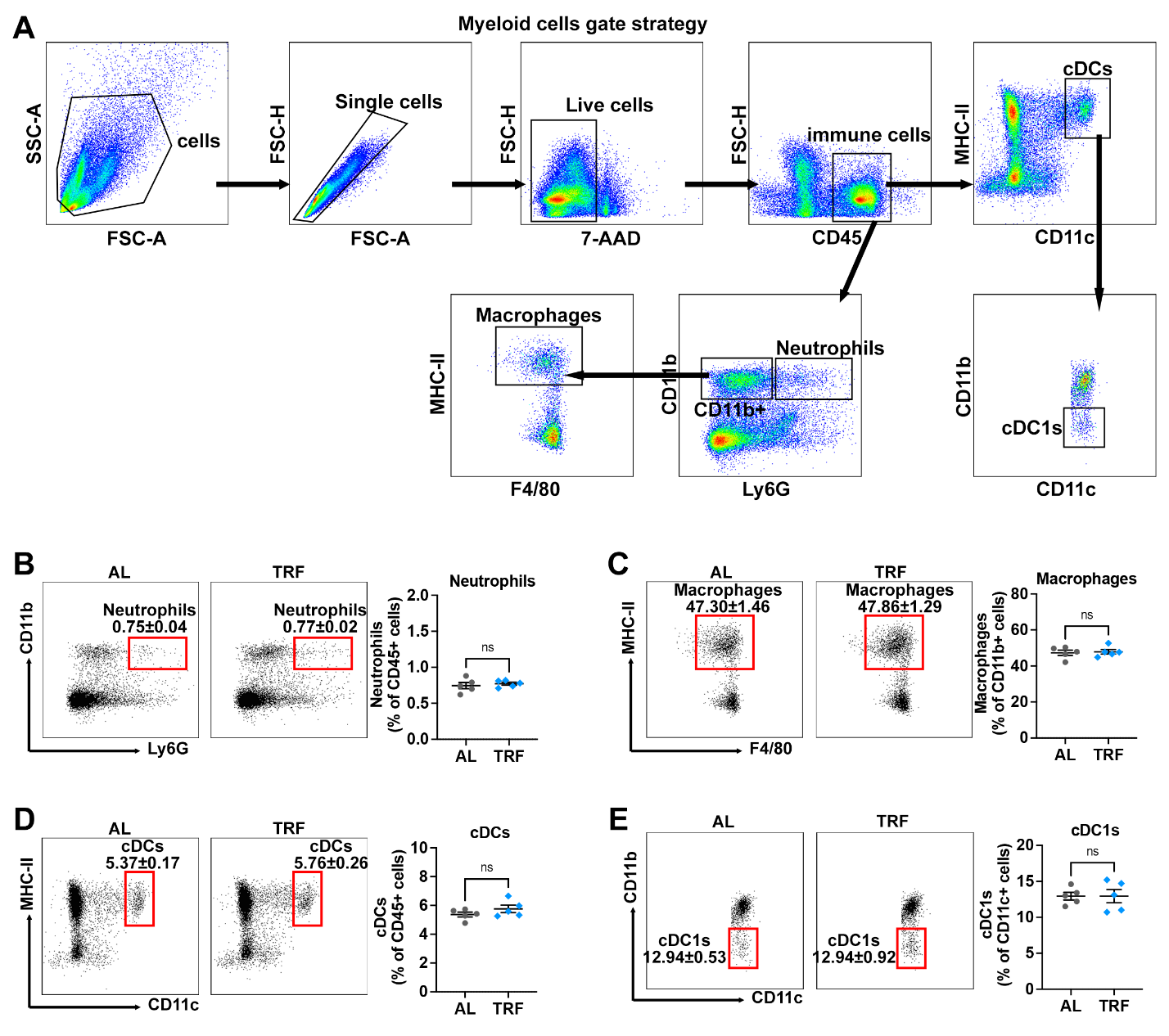


**Figure S4.** Myeloid cells in the intestinal lamina propria. (A) Gate strategies of myeloid cells in the intestinal lamina propria. (B) Dot plots and frequenct of neutrophils. (C) Dot plots and frequenct of macrophages. (D-E) Dot plots and frequenct of cDCs and cDC1s. Data are presented as mean ± SEM. (n=5 per group; ns, no significance).


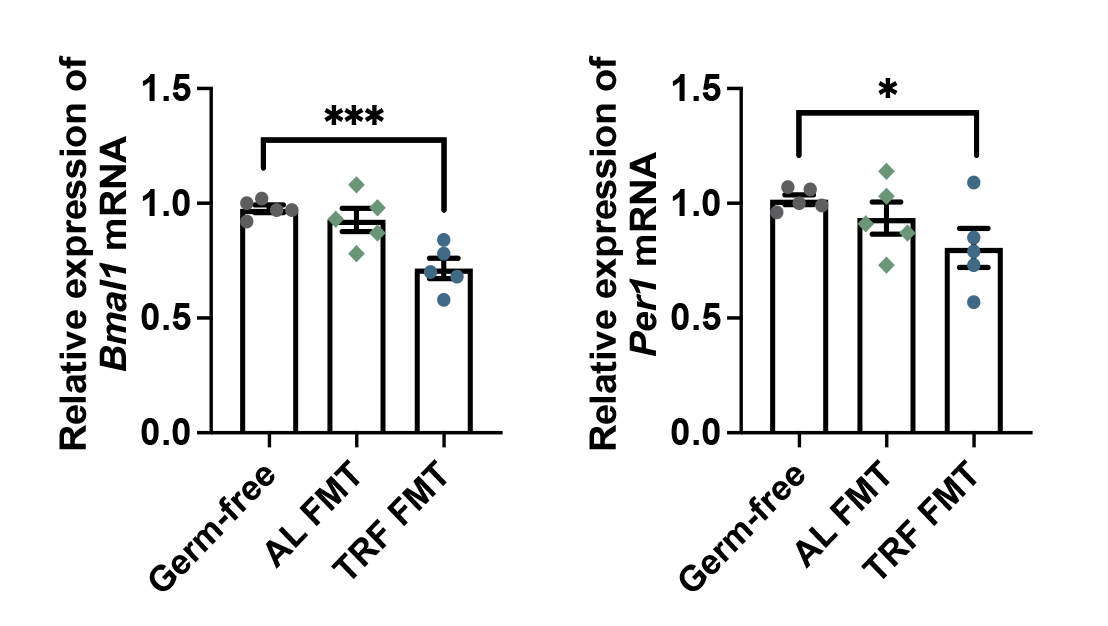


**Figure S5.** The mRNA expression of circadian genes *Bmal1* and *Per1* in the small intestine of FMT mice. Data are presented as mean ± SEM. (n=5 per group, * *P*<0.05; *** *P*< 0.001).

**Table S1.** Antibodies for flow cytometry

| **Antibody** | **Company** | **Cat No.** |
| --- | --- | --- |
| 7-AAD | BD Pharmingen | 559925 |
| Ms CD45 BV510 | BD Pharmingen | 564279 |
| Ms CD3e PE-Cy7 | BD Pharmingen | 552774 |
| Ms CD4 FITC | BD Pharmingen | 557307 |
| Ms IL-17a BV421 | BD Pharmingen | 566286 |
| Ms CD8a APC | BD Pharmingen | 561093 |
| Ms CD11b FITC | BD Pharmingen | 553310 |
| Ms Ly6G PE-Cy7 | BD Pharmingen | 560601 |
| Ms CD11c APC | BD Pharmingen | 550261 |
| Ms IA/IE BV605 | BD Pharmingen | 563413 |
| Ms F4/80 PE | BD Pharmingen | 565410 |
| Ms Foxp3 PE | Invitrogen | 12-5773-82 |

**Table S2.** Primer sequences for qRT-PCR

| **Genes** | **Forward (5′-3′)** | **Reverse (5′-3′)** |
| --- | --- | --- |
| *Gapdh* | ATCATCCCTGCATCCACT | TCTTCAGGGCTTTCTCGTTC |
| *Ocn* | GAGGGCAATAAGGTAGTGA ACAGA | AAGCCATACTGGTTTGATAGCTCG |
| *Runx* | TTCTCCAACCCACGAATGCAC | CAGGTACGTGTGGTAGTGAG |
| *Alp* | ACCACCACGAGAGTGAACCA | CGTTGTCTGAGTACCAGTCCC |
| *Trap* | AGTAAGGGCTGGGAAGT | AGCGACAAGAGGTTCCAG |
| *Ctsk* | CTTCCAATACGTGCAGCAGA | TCTTCAGGGCTTTCTCGTTC |
| *Fos* | GCGAGCAACTGAGAAGAC | TTGAAACCCGAGAACATC |
| *Mmp9* | CGTGTCTGGAGATTCGACTTGA | TTGGAAACTCACACGCCAGA |
| *Nfatc1* | CAACGCCCTGACCACCGATAG | GGCTGCCTTCCGTCTCATAGT |
| *Il17a* | TGACGCCCACCTACAACATC | CATCATGCAGTTCCGTCAGC |
| *Acp5* | CACTCCCACCCTGAGATTTGT | CCCCAGAGACATGATGAAGTCA |
| *Bpgm* | GGACCAGAAACTTAACAACGACG | CATAGTGACGCTCATTCAGACG |
| *Fbxo7* | CCCACGTTGGGGTTCAGTTC | TCCTGGAGTGAGGAATGCTCT |
| *Rankl* | AGCCGAGACTACGGCAAGTA | AAAGTACAGGAACAGAGCGATG |
| *Opg* | CCTTGCCCTGACCACTCTTAT | CACACACTCGGTTGTGGGT |
| *Tgfb1* | CTTCAATACGTCAGACATTCGGG | GTAACGCCAGGAATTGTTGCTA |
| *Il10* | CTTACTGACTGGCATGAGGATCA | GCAGCTCTAGGAGCATGTGG |
| *Bmal1* | CGCCGCTCTCTGTTCTGTAG | GTGTCGAGAAACGTACTCCATAG |
| *Per1* | GAGGGACGAGTCTTACCATGA | AATAGACGCCACCTTGGTTTG |
